# Intraperitoneal, but not retroperitoneal, visceral adipose tissue is associated with diabetes mellitus: a cross-sectional, retrospective pilot analysis

**DOI:** 10.1186/s13098-020-00612-5

**Published:** 2020-11-25

**Authors:** Muhei Tanaka, Hiroshi Okada, Yoshitaka Hashimoto, Muneaki Kumagai, Hiromi Nishimura, Michiaki Fukui

**Affiliations:** 1 Department of Endocrinology and Metabolism, Kyoto Prefectural University of Medicine, Graduate School of Medical Science, 465 Kajii-cho, Kawaramachi-Hirokoji, Kamigyo-ku, Kyoto, 602-8566 Japan; 2 Department of Internal Medicine, Matsushita Memorial Hospital, Osaka, Japan; 3 Medical Corporation Soukenkai, Nishimura Clinic, Kyoto, Japan

**Keywords:** Intraperitoneal visceral adipose tissue, Retroperitoneal visceral adipose tissue, Diabetes mellitus, Subcutaneous adipose tissue, Insulin resistance

## Abstract

**Aim:**

Diabetes mellitus (DM) is associated with adverse outcomes, and visceral adipose tissue (VAT), classified into intraperitoneal VAT (IVAT) and retroperitoneal VAT (RVAT), is associated with insulin resistance. This study aimed to evaluate the association of IVAT and RVAT with the prevalence or incidence of DM.

**Methods:**

In this cross-sectional, retrospective, cohort study, the prevalence and incidence of DM was analyzed in 803 and 624 middle-aged Japanese participants, respectively. The cross-sectional area of the abdominal adipose tissue was evaluated from an unenhanced computed tomography scan at the third lumbar vertebrae, and the IVAT or RVAT was analyzed using specialized software. The areas were normalized for the square value of the participants’ height in meters and described as the IVAT or RVAT area index.

**Results:**

The IVAT area index (adjusted odds ratio [OR], 1.04; 95% confidence intervals [CI], 1.02–1.07, per 1.0 cm^2^/m^2^) or IVAT/RVAT area ratio (1.89; 1.23–2.85, per 1.0) was independently associated with the prevalence of DM, whereas the RVAT area index was not. During a follow-up (mean) of 3.7 years, 30 participants were diagnosed with DM. The IVAT area index (adjusted hazards ratio [HR], 1.02; 95% CI 1.003–1.04, per 1.0 cm^2^/m^2^) or IVAT/RVAT area ratio (2.25; 1.40–3.43, per 1.0) was independently associated with the incidence of DM, whereas the RVAT area index was not.

**Conclusions:**

IVAT, but not RVAT, is associated with the prevalence or incidence of DM.

## Background

It is already well established in the literature that diabetes mellitus (DM) have a higher risk of atherosclerosis and cardiovascular disease [1, 2]. There exists a close relationship between visceral adipose tissue (VAT) and, both, peripheral and hepatic insulin resistance in patients with type 2 DM [3].

Obesity includes many different anatomical, physiological and pathological phenotypes, and both total adiposity and regional fat distribution influence metabolism [4–6]. Abdominal adipose tissue can be differentiated into subcutaneous adipose tissue (SAT) and VAT, which have different functions in lipid and glucose metabolism [7]. The VAT volumes are associated with the metabolic consequences of obesity [8, 9], although investigations concerning SAT have revealed controversial results [8]. Moreover, SAT and VAT can be further distinguished as deep and superficial SAT and intraperitoneal and retroperitoneal VAT, respectively [9–12]; whereas the deep and superficial SAT have different functions [13–15], the difference between the intraperitoneal and retroperitoneal VAT has not been examined.

Computed tomography (CT) scanning is the gold standard investigational method to analyze the SAT and VAT [16, 17]. Recently, a study showed that the site-specific measurement of abdominal adipose tissue (intraperitoneal and retroperitoneal VAT, or deep and superficial SAT) evaluated by CT scanning demonstrated high repeatability [9].

Therefore, in this study, we investigated whether the intraperitoneal and retroperitoneal VAT areas, as evaluated by CT, were associated with the prevalence or incidence of DM in middle-aged Japanese participants.

## Materials and methods

### Participants and study design

**Fig. 1:**
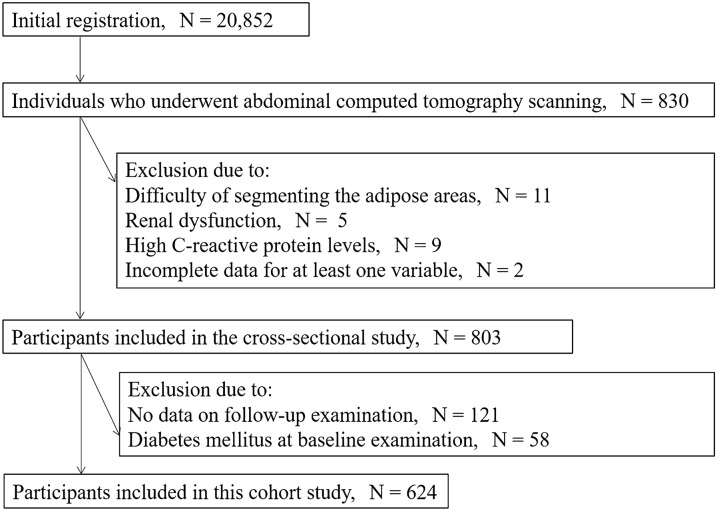
Flowchart of the screening and inclusion and exclusion of study participants

The Nishimura Health Survey is an ongoing cohort investigation of risk factors for chronic diseases including metabolic syndrome, hypertension, chronic kidney disease, non-alcoholic fatty liver disease, and diabetes mellitus [18–21]. The Nishimura Clinic (Kyoto, Japan) provides regular health check-up for employees of various companies. A cross-sectional study as well as a retrospective cohort study with mean and median follow-up durations of 3.7 and 4.0 years were performed to evaluate the site-specific measurement of abdominal fat and its correlation with the prevalence or incidence of DM. From the 20,852 individuals who underwent physical health checkups from April 2013 to March 2018, 830 individuals who had undergone abdominal CT scanning were evaluated for study inclusion. In Japan, yearly routine examination for employees is legally mandated, and all or most of the costs for the health check-up are usually paid by their employers. Although abdominal CT scanning was not part of the basic examinations, it was performed on request of 830 individuals. Individuals were excluded in case of incomplete data, difficulty with segmenting the adipotic areas (because the fascia separating the areas could not be visualized), renal dysfunction, and high C-reactive protein (CRP) levels (Fig. 1), because active infection, systemic inflammatory processes, and kidney dysfunction can also affect glucose metabolism. Additionally, participants were excluded from this cohort study if they had no data on follow-up examinations and if they were diagnosed with DM at the baseline examination. Finally, in this cross-sectional, retrospective, cohort study, the prevalence and incidence of DM was analyzed in 803 and 624 participants, and 30 participants were newly diagnosed with DM during the study period. All procedures were approved by the Local Research Ethics Committee of the Kyoto Prefectural University of Medicine (ERB-C-1017–1) and conducted in accordance with the Declaration of Helsinki. All study participants provided informed consent for study participation.

### Data collection and measurements, and definitions

Demographic data and biomarkers were assessed as described previously [18–21], and the biomarkers evaluated included fasting plasma glucose (FPG), triglycerides, low-density lipoprotein cholesterol (LDL-C), high-density lipoprotein cholesterol (HDL-C), creatinine, and CRP levels. Smoking was defined as current tobacco use. Alcohol drinking habits were evaluated based on the amount and frequency of alcoholic beverage intake during the past month, which was converted to daily alcohol intake. Subjects were classified as positive for alcohol drinking if the consumption exceeded 30 g/ day and 20 g/day for men and women respectively. When subjects had performed any kind of physical activity for at least 30 min/day regularly, they were categorized as regular exercisers. According to the American Diabetes Association, diabetes mellitus was defined as having a HbA1c level ≥ 6.5% (48 mmol/mol), FPG level ≥ 126 mg/dL (7.0 mmol/L), in addition to a medical history of diabetes, or current use of antidiabetic agents [22]. Hypertension was defined as a systolic blood pressure > 140 mmHg, or a diastolic blood pressure > 90 mmHg, in addition to a medical history of hypertension, or current use of antihypertensive agents. Dyslipidemia was defined as either or a combination of LDL-C ≥ 4.14 mmol/L (160 mg/dL), HDL-C < 1.03 mmol/L (40 mg/dL), or triglyceride level ≥ 1.69 mmol/L (150 mg/dl), in addition to a medical history of dyslipidemia, or current use of lipid-lowering agents. High CRP level was defined as values > 95.2 nmol/L (10 mg/L). Renal dysfunction was defined based on a serum creatinine threshold > 106.1 μmol/L (1.2 mg/dL).

### Assessment of abdominal adipose tissue area

The CT settings and analysis software have been described in previous studies [18–21]. The cross-sectional area of the abdominal adipose tissue was evaluated from an unenhanced CT scan at the third lumbar vertebrae, and was semi-automatically calculated by a well-trained technician who was blinded to the participant’s identity and clinical presentation. The VAT (including IVAT, and RVAT) and SAT (including DSAT, and SSAT) were identified and quantified using Hounsfield unit (HU) thresholds of − 150 to − 50 and − 190 to − 30 HU, respectively [23]. The intraperitoneal and retroperitoneal VAT (IVAT, and RVAT), and deep and superficial SAT (DSAT, and SSAT) were identified and quantified according to a method that was reported in the literature [9]. The cross-sectional areas were normalized for the square of the participants’ height in meters and described as the VAT, SAT, IVAT, RVAT, DSAT, or SSAT area indices. Additionally, we calculated VAT/SAT, IVAT/RVAT, and DSAT/SSAT ratios as the area ratios of VAT area index divided by SAT area index, the area ratio of IVAT area index divided by RVAT area index, and the area ratio of DSAT area index divided by SSAT area index, respectively. The intraclass correlation coefficients for each VAT, SAT, IVAT, RVAT, DSAT, or SSAT area indices from 200 random subset samples were all > 0.94.

### Study endpoints

This study has been designed to investigate potential different associations between DM and IVAT and RVAT. We particularly focused on middle-aged Japanese participants. The primary end-point was to determine whether IVAT and RVAT have different associations with the prevalence of DM. The secondary endpoint was to determine whether IVAT is able to predict incidence of DM.

### Statistical analysis

No formal sample size calculation was performed, because previous studies were not available regarding the relationship between DM and IVAT or RVAT. Continuous parameters are presented as mean ± standard deviation, and categorical parameters are presented as number (percentage). A skewed variable, such as CRP, was presented as median (interquartile range). The Student’s *t*-test or chi-square test was performed to assess the significance of differences between the two groups. The Pearson correlation coefficient was used to measure the strength of a linear association between two continuous parameters. Originally showing a skewed distribution, CRP level was log transformed. Logistic regression analysis was used to assess the association of the parameters of abdominal adipose tissue area with the prevalence of DM. We have evaluated the coefficient of determination between all independent variables to detect multicollinearity, and all values did not exceeds 0.7. The adjusted odds ratios (ORs) of each parameter of the abdominal adipose tissue area for the prevalence of DM were calculated; to avoid excessive overfitting, the following parameters were used simultaneously as independent variables: Model 1: age, sex, body mass index, and each parameter for abdominal adipose tissue area; Model 2: Model 1 plus prevalence of hypertension, and dyslipidemia; and Model 3: Model 1 plus C-reactive protein, and creatinine levels. Additionally, to analyze the IVAT, RVAT, DSAT, and SSAT area indices simultaneously, the adjusted ORs for the prevalence of DM were calculated; to avoid excessive overfitting, the following parameters were used simultaneously as independent variables: age, sex, body mass index, and the IVAT, RVAT, DSAT and SSAT area indices. Multiple regression analysis was performed to explore the associations between the CRP levels and the parameters of abdominal adipose tissue area in the cross-sectional study. Multiple Cox regression analyses were performed to calculate the hazards ratio (HR) of each parameter of the abdominal adipose tissue area for incident DM. To avoid excessive overfitting, the following parameters were simultaneously used as independent variables: Model 1: age, sex, fasting plasma glucose at baseline examination, and each parameter for abdominal adipose tissue area, Model 2: Model 1 plus body mass index. To compare which abdominal fat compartment is more informative compared other compartment and to its ratios, we have conducted both receiver operator characteristic (ROC) curves and Bayesian Information Criterions analyses. The ROC analyses were performed to calculate the area under the ROC curves (AUC) of the IVAT, RVAT, DSAT, and SSAT area indices, and VAT/SAT, IVAT/RVAT and DSAT/SSAT area ratios for the prevalence of DM. We also compared the AUC between the two groups. A *P*-value of < 0.05 was considered significant. Statistical analyses were performed using the JMP version 11.0 software (SAS Institute Inc., Cary, NC, USA).

## Results

In the cross-sectional study, there were significant differences between the participants with and without DM in regard to the total adipose tissue area index; VAT, SAT, IVAT, RVAT, DSAT, and SSAT area indices; and VAT/SAT, IVAT/RVAT and DSAT/SSAT area ratios (Table 1). The participants with DM were older, and had a higher body mass index. Therefore, we have evaluated the association between these variables and adipose tissue parameters. Age was positively associated with the total adipose tissue area index; VAT, IVAT, RVAT, and DSAT area indices; and VAT/SAT, IVAT/RVAT and DSAT/SSAT area ratios. Body mass index was positively associated with the total adipose tissue area index; VAT, SAT, IVAT, RVAT, DSAT, and SSAT area indices; and VAT/SAT, IVAT/RVAT and DSAT/SSAT area ratios. Male participants had higher VAT, IVAT, and RVAT area indices; and VAT/SAT, IVAT/RVAT and DSAT/SSAT area ratios, and had lower SAT, DSAT, and SSAT area indices.

**Table 1:** Clinical characteristics of study participants in this cross-sectional and retrospective cohort studies

	Cross-sectional study			Retrospective cohort study		
	Prevalence of DM (−)	Prevalence of DM (+)	*P*	Incidence of DM (−)	Incidence of DM (+)	*P*
n	745	58		594	30	
Age (years)	50.6 ± 10.6	58.6 ± 9.5	< 0.0001	50.8 ± 10.6	54.4 ± 9.8	0.0703
Male	458 (61.5)	48 (82.8)	0.0012	370 (62.3)	24 (80.0)	0.0498
Body mass index (kg/m^2^)	22.6 ± 3.4	25.8 ± 4.9	< 0.0001	22.6 ± 3.4	25.7 ± 4.5	< 0.0001
Family history of diabetes mellitus	102 (13.7)	22 (37.9)	< 0.0001	516 (86.9)	25 (83.3)	0.5780
Current smoker	120 (16.1)	18 (31.0)	0.0037	500 (84.2)	24 (80.0)	0.5431
Regular exercise	195 (26.2)	25 (43.1)	0.0054	434 (73.1)	25 (83.3)	0.2134
Alcohol drinking habit	163 (21.9)	13 (22.4)	0.9245	464 (78.1)	22 (73.3)	0.5381
Hypertension	192 (25.8)	31 (53.5)	< 0.0001	441 (74.2)	19 (63.3)	0.1854
Dyslipidemia	220 (29.5)	35 (60.3)	< 0.0001	420 (70.7)	19 (63.3)	0.3882
C-reactive protein^a^ (nmol/L)	1.9 (1.9–7.6)	7.6 (4.3–24.8)	< 0.0001	1.9 (1.9–6.9)	8.6 (1.9–11.7)	0.0020
Creatinine (μmol/L)	68.3 ± 13.5	72.6 ± 13.9	0.0208	68.5 ± 13.6	74.4 ± 11.0	0.0195
Fasting plasma glucose (mmol/L)	5.3 ± 0.5	8.0 ± 2.1	< 0.0001	5.3 ± 0.4	6.1 ± 0.5	< 0.0001
Total adipose tissue area index (cm^2^/m^2^)	79.4 ± 40.2	123.3 ± 59.0	< 0.0001	77.8 ± 39.9	111.1 ± 37.0	< 0.0001
VAT area index(cm^2^/m^2^)	31.5 ± 21.2	58.6 ± 24.9	< 0.0001	30.7 ± 21.0	51.2 ± 19.5	< 0.0001
IVAT area index(cm^2^/m^2^)	19.3 ± 14.6	38.3 ± 17.7	< 0.0001	18.8 ± 14.4	33.1 ± 14.2	< 0.0001
RVAT area index(cm^2^/m^2^)	12.2 ± 7.5	20.0 ± 9.4	< 0.0001	12.0 ± 7.4	18.1 ± 7.1	< 0.0001
SAT area index(cm^2^/m^2^)	47.9 ± 24.7	64.6 ± 43.8	< 0.0001	47.1 ± 24.3	59.9 ± 27.0	0.0053
DSAT area index (cm^2^/m^2^)	27.9 ± 15.1	40.6 ± 26.3	< 0.0001	27.5 ± 15.1	36.7 ± 15.3	0.0012
SSAT area index (cm^2^/m^2^)	20.0 ± 11.4	24.4 ± 20.4	0.0090	19.6 ± 11.2	23.2 ± 14.3	0.0910
VAT/SAT area ratio	0.7 ± 0.4	1.1 ± 0.5	< 0.0001	0.7 ± 0.4	1.0 ± 0.4	0.0001
IVAT/RVAT area ratio	1.6 ± 0.7	2.0 ± 0.6	< 0.0001	1.5 ± 0.7	1.9 ± 0.7	0.0017
DSAT/SSAT area ratio	1.5 ± 0.6	1.8 ± 0.6	0.0001	1.5 ± 0.6	1.8 ± 0.7	0.0107

Continuous variables are presented as mean ± 1 standard deviation, or median (interquartile range), and categorical variables are presented as number (percentage)

*DM* diabetes mellitus, *VAT* visceral adipose tissue, *IVAT* intraperitoneal VAT, *RVAT* retroperitoneal VAT, *SAT* subcutaneous adipose tissue, *DSAT* deep SAT, *SSAT* superficial SAT. ^a^Values were analyzed after log transformation

In the adjusted model 3, the VAT area index (OR, 1.03; 95% confidence interval (CI), 1.01–1.05, per 1.0 cm^2^/m^2^) or the IVAT area index (OR, 1.04; 95% CI 1.02–1.07, per 1.0 cm^2^/m^2^) was independently associated with the prevalence of DM, whereas the RVAT area index was not (Table 2). Moreover, in the same model, the VAT/SAT area ratio (OR, 3.21; 95% CI 1.49–6.83, per 1.0) or the IVAT/RVAT area ratio (OR, 1.89; 95% CI 1.23–2.85, per 1.0) was independently associated with the prevalence of DM. In the adjusted model 1 and 2, similar results were obtained. After adjusting for age, sex, body mass index and family history of diabetes mellitus, the IVAT (OR, 1.05; 95% CI 1.02–1.08, per 1.0 cm^2^/m^2^) or RVAT (OR, 1.05; 95% CI 1.002–1.10, per 1.0 cm^2^/m^2^) area index was independently associated with the prevalence of DM. Besides, after adjusting for age, sex, body mass index, smoking status, and exercise habits, the IVAT (OR, 1.05; 95% CI 1.02–1.07, per 1.0 cm^2^/m^2^) or RVAT (OR, 1.05; 95% CI 1.002–1.10, per 1.0 cm^2^/m^2^) area index was independently associated with the prevalence of DM. However, in the adjusted model 1.2 and 3, the RVAT area index was not independently associated with the prevalence of DM.

**Table 2:** Adjusted odds ratio and 95% confidence interval of each abdominal adipose tissue area parameter for the prevalence of diabetes mellitus

	Model 1	Model 2	Model 3
Total adipose tissue area index, per 1.0 cm^2^/m^2^	1.02 (1.01–1.04)*	1.02 (1.01–1.04)*	1.02 (1.01–1.04)*
VAT area index, per 1.0 cm^2^/m^2^	1.03 (1.01–1.05)*	1.03 (1.01–1.05)*	1.03 (1.01–1.05)*
IVAT area index, per 1.0 cm^2^/m^2^	1.04 (1.02–1.07)*	1.04 (1.01–1.06)*	1.04 (1.02–1.07)*
RVAT area index, per 1.0 cm^2^/m^2^	1.04 (0.99–1.09)	1.03 (0.98–1.08)	1.03 (0.99–1.08)
SAT area index, per 1.0 cm^2^/m^2^	1.01 (0.99–1.03)	1.01 (0.99–1.04)	1.01 (0.99–1.03)
DSAT area index, per 1.0 cm^2^/m^2^	1.02 (0.99–1.05)	1.02 (0.99–1.05)	1.01 (0.99–1.04)
SSAT area index, per 1.0 cm^2^/m^2^	1.02 (0.98–1.05)	1.02 (0.98–1.05)	1.02 (0.98–1.05)
VAT/SAT area ratio, per 1.0	3.30 (1.55–6.95)*	2.95 (1.35–6.37)*	3.21 (1.49–6.83)*
IVAT/RVAT area ratio, per 1.0	1.94 (1.27–2.91)*	1.86 (1.20–2.83)*	1.89 (1.23–2.85)*
DSAT/SSAT area ratio, per 1.0	1.07 (0.67–1.59)	1.09 (0.67–1.65)	1.01 (0.62–1.52)

The following parameters were simultaneously used as independent variables: Model 1: age, sex, body mass index, and each parameter for abdominal adipose tissue area, Model 2: Model 1 plus prevalence of hypertension, and dyslipidemia, Model 3: Model 1 plus C-reactive protein, and creatinine levels

*VAT* visceral adipose tissue, *IVAT* intraperitoneal VAT, *RVAT* retroperitoneal VAT, *SAT* subcutaneous adipose tissue, *DSAT* deep SAT, *SSAT* superficial SAT

* *P* < 0.05

When the IVAT, RVAT, DSAT, and SSAT area indices were used simultaneously as independent variables, the IVAT area index was independently associated with the prevalence of DM, whereas the RVAT, DSAT, and SSAT area indices were not (Table 3).

**Table 3:** Adjusted odds ratios and 95% confidence intervals for the prevalence of diabetes mellitus

	Odds ratios and 95% confidence intervals
IVAT area index, per 1.0 cm^2^/m^2^	1.04 (1.02–1.08)*
RVAT area index, per 1.0 cm^2^/m^2^	1.00 (0.94–1.05)
DSAT area index, per 1.0 cm^2^/m^2^	1.01 (0.98–1.04)
SSAT area index, per 1.0 cm^2^/m^2^	1.02 (0.99–1.06)

The following parameters were simultaneously used as independent variables: age, sex, body mass index, and IVAT, RVAT, DSAT and SSAT area indexes

*IVAT* intraperitoneal visceral adipose tissue, *RVAT* retroperitoneal visceral adipose tissue, *DSAT* deep subcutaneous adipose tissue, *SSAT* superficial subcutaneous adipose tissue

* *P* < 0.05

In the cross-sectional study, multiple regression analysis showed that the IVAT area index (*β* = 0.178, *P* = 0.0048), but not the RVAT area index (*β* = 0.083, *P* = 0.1815), was independently associated with CRP levels when the following parameters were simultaneously used as independent variables: age, sex, IVAT, RVAT, DSAT, and SSAT area indices.

**Fig. 2:**
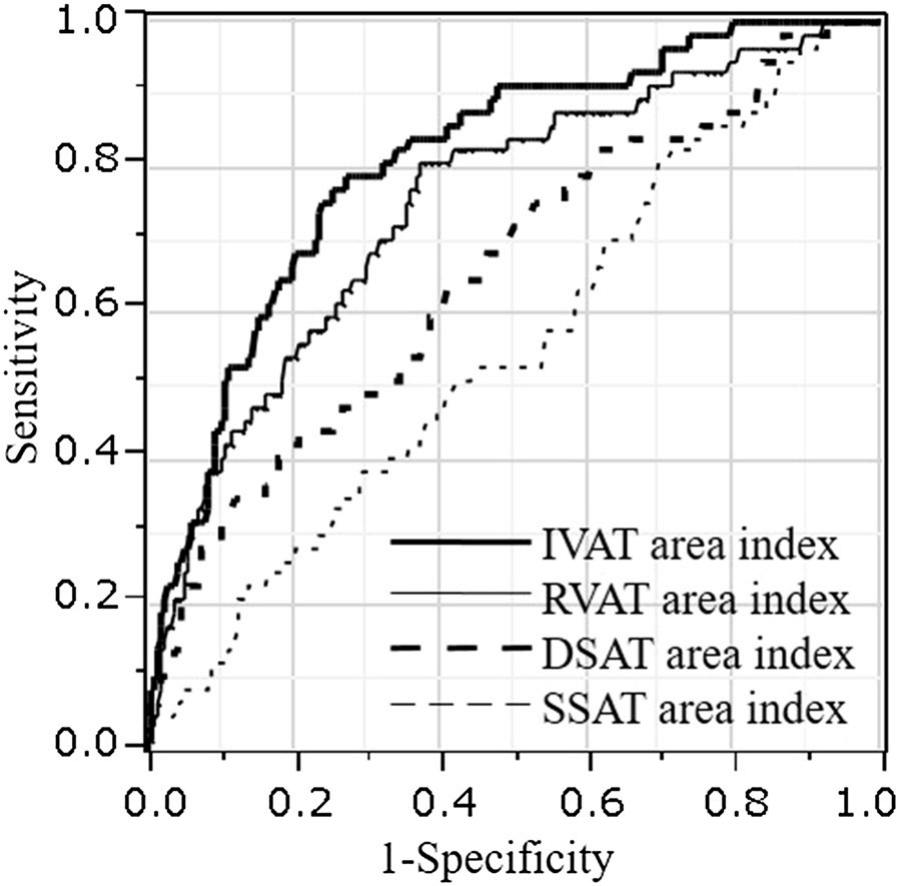
Area under the receiver operating characteristic curves (AUC) of IVAT, RVAT, DSAT, and SSAT area indices for the prevalence of diabetes mellitus. *VAT* visceral adipose tissue, *IVAT* intraperitoneal VAT, *RVAT* retroperitoneal VAT, *SAT* subcutaneous adipose tissue, *DSAT*, deep SAT, *SSAT* superficial SAT, *CI* confidence interval

To compare which abdominal fat compartment is more informative compared other compartment and to its ratios, we performed the following statistical analyses. The AUC of the IVAT area index for the prevalence of DM was larger than that of the RVAT, DSAT, or SSAT area index (Tables 4, 5 and Fig. 2). In the variables such as IVAT, and DSAT area indices, and IVAT/RVAT and DSAT/SSAT area ratios, IVAT/RVAT area ratio was selected by Bayesian Information Criterions.

**Table 4:** Receiver operating characteristic curve analyses of each abdominal adipose tissue area parameter for identifying the prevalence of diabetes mellitus

	AUC	*P*	Cutoff value	Sensitivity	Specificity
Total adipose tissue area index (cm^2^/m^2^)	0.745	< 0.0001	99.4	0.667	0.728
VAT area index(cm^2^/m^2^)	0.804	< 0.0001	46.9	0.741	0.779
IVAT area index(cm^2^/m^2^)	0.809	< 0.0001	26.9	0.790	0.729
RVAT area index(cm^2^/m^2^)	0.751	< 0.0001	13.7	0.807	0.628
SAT area index(cm^2^/m^2^)	0.617	< 0.0001	37.5	0.828	0.380
DSAT area index (cm^2^/m^2^)	0.657	< 0.0001	25.3	0.351	0.882
SSAT area index (cm^2^/m^2^)	0.555	0.0181	13.1	0.825	0.286
VAT/SAT area ratio	0.725	< 0.0001	0.709	0.754	0.600
IVAT/RVAT area ratio	0.705	< 0.0001	1.760	0.684	0.667
DSAT/SSAT area ratio	0.675	0.0009	1.579	0.684	0.644

*AUC* area under the curve, *VAT* visceral adipose tissue, *IVAT* intraperitoneal VAT, *RVAT* retroperitoneal VAT, *SAT* subcutaneous adipose tissue, *DSAT* deep SAT, *SSAT* superficial SAT

**Table 5:** Pairwise comparison of AUCs of each abdominal adipose tissue area parameter for identifying the prevalence of diabetes mellitus (*P*-values)

	Total adipose tissue area index	VAT area index	IVAT area index	RVAT area index	SAT area index	DSAT area index	SSAT area index	VAT/SAT area ratio	IVAT/RVAT area ratio	DSAT/SSAT area ratio
Total adipose tissue area index	–	0.0003	0.0003	0.7586	< 0.0001	< 0.0001	< 0.0001	0.6712	0.3940	0.1443
VAT area index	–	–	0.2462	< 0.0001	< 0.0001	< 0.0001	< 0.0001	0.0248	0.0248	0.0050
IVAT area index	–	–	–	0.0018	< 0.0001	< 0.0001	< 0.0001	0.0105	0.0052	0.0026
RVAT area index	–	–	–	–	0.0002	0.0065	< 0.0001	0.4895	0.3832	0.1053
SAT area index	–	–	–	–	–	0.2895	0.0007	0.0760	0.0940	0.2895
DSAT area index	–	–	–	–	–	–	0.0008	0.2405	0.3502	0.6813
SSAT area index	–	–	–	–	–	–	–	0.0050	0.0028	0.0573
VAT/SAT area ratio	–	–	–	–	–	–	–	–	0.6534	0.2780
IVAT/RVAT area ratio	–	–	–	–	–	–	–	–	–	0.5692
DSAT/SSAT area ratio	–	–	–	–	–	–	–	–	–	–

*AUC* area under the curve, *VAT* visceral adipose tissue, *IVAT* intraperitoneal VAT, *RVAT* retroperitoneal VAT, *SAT* subcutaneous adipose tissue, *DSAT* deep SAT, *SSAT* superficial SAT

The incidence of DM across 2324 person-years was 13 per 1000 person-years (95% CI 9–18) in 624 participants. In Cox regression analyses, the c-statistics for the following parameters was greater than 0.7: body mass index, 0.752; fasting plasma glucose, 0.885; VAT area index, 0.769; IVAT area index, 0.800, RVAT area index, 0.754; SAT area index, 0.704; DSAT area index, 0.736. In the adjusted model 1, the IVAT area index (HR, 1.02; 95% CI 1.003–1.04, per 1.0 cm^2^/m^2^) or the IVAT/RVAT area ratio (HR, 2.25; 95% CI 1.40–3.43, per 1.0) was independently associated with the incidence of DM, whereas the RVAT area index was not (Table 6). In the adjusted model 2, only IVAT/RVAT area ratio (HR, 2.22; 95% CI 1.33–3.47, per 1.0) was independently associated with the incidence of DM.

**Table 6:** Adjusted hazard ratio and 95% confidence interval of each abdominal adipose tissue area parameter for incident diabetes mellitus

	Model 1	Model 2
Total adipose tissue area index, per 1.0 cm^2^/m^2^	1.01 (1.00–1.01)	1.00 (0.99–1.02)
VAT area index, per 1.0 cm^2^/m^2^	1.02 (1.00–1.03)	1.01 (0.99–1.03)
IVAT area index, per 1.0 cm^2^/m^2^	1.02 (1.003–1.04)*	1.02 (0.99–1.05)
RVAT area index, per 1.0 cm^2^/m^2^	1.02 (0.97–1.07)	0.99 (0.92–1.05)
SAT area index, per 1.0 cm^2^/m^2^	1.01 (1.00–1.02)	0.99 (0.96–1.02)
DSAT area index, per 1.0 cm^2^/m^2^	1.01 (0.99–1.03)	0.98 (0.95–1.02)
SSAT area index, per 1.0 cm^2^/m^2^	1.02 (0.99–1.06)	1.00 (0.94–1.05)
VAT/SAT area ratio, per 1.0	2.30 (0.86–5.59)	2.56 (0.92–6.60)
IVAT/RVAT area ratio, per 1.0	2.25 (1.40–3.43)*	2.22 (1.33–3.47)*
DSAT/SSAT area ratio, per 1.0	0.96 (0.52–1.55)	0.89 (0.48–1.47)

The following parameters were simultaneously used as independent variables: Model 1: age, sex, fasting plasma glucose at baseline examination, and each parameter for abdominal adipose tissue area, Model 2: Model 1 plus body mass index

*VAT* visceral adipose tissue, *IVAT* intraperitoneal VAT, *RVAT* retroperitoneal VAT, *SAT* subcutaneous adipose tissue, *DSAT* deep SAT, *SSAT* superficial SAT

* *P* < 0.05

## Discussion

This study has revealed two primary findings. First, the IVAT or RVAT area index, or IVAT/RVAT area ratio was independently associated with the prevalence of DM. However, the AUC of the IVAT area index for the prevalence of DM was larger than that of the RVAT area index, or IVAT/RVAT area ratio. Moreover, even after the simultaneous inclusion of the IVAT, RVAT, DSAT, and SSAT area indices in the multivariate logistic regression analysis, the IVAT area index was independently associated with the prevalence of DM, whereas the RVAT area index was not. Second, the IVAT area index or the IVAT/RVAT area ratio was independently associated with the incidence of DM, whereas the RVAT area index was not. Moreover, in the adjustment model including body mass index, only IVAT/RVAT area ratio was independently associated with the incidence of DM, whereas the IVAT or RVAT area index was not. To the best of our knowledge, this is the first study to demonstrate the independent association of IVAT, or IVAT/RVAT area ratio, but not RVAT, with the prevalence or incidence of DM.

This is the first study to evaluate the association between DM and IVAT area based on CT evaluations. A few studies have identified an association between insulin resistance and the IVAT and RVAT areas based on findings from magnetic resonance imaging (MRI) [11, 12]. However, the results of those studies have been inconsistent. The IVAT area evaluated by MRI was a major predictor of peripheral and hepatic insulin action in 57 participants with obesity, whereas the RVAT area was not [12]. Conversely, no difference between IVAT and RVAT by MRI on insulin resistance was reported from a study among 89 obese men [11]. On the other hand, our study demonstrated the distinct associations of IVAT and RVAT with glucose metabolism, although there was a difference in the assessment tool of abdominal adipose tissue area. Indeed, unlike previous studies, our study directly compared the effects of IVAT and RVAT on glucose metabolism. Moreover, a larger number of participants were evaluated than in previous studies. Our study also demonstrated that the IVAT/RVAT area ratio was important for the incidence of DM, even after adjusted by body mass index.

The insulin action was negatively associated with the VAT area, because increased delivery of free fatty acids (FFAs) in insulin sensitive tissues significantly impairs insulin action [11, 24, 25]. In particular, the adverse effects associated with VAT may be related to the portally drained FFAs originating from IVAT [25, 26]. Mice that received a fat transplant which drained into the portal vein had higher blood glucose levels after a glucose load than mice that received a fat transplant that drained into the inferior vena cava or the sham-operated mice [27]. The portal theory is a possible hypothesis of the association between glucose metabolism and the IVAT [25, 27]. The link between the VAT and insulin resistance involves the close association with fatty liver diseases. The IVAT relates to portal circulation, whereas the RVAT relates to the systemic circulation. The fatty tissue, which was drained into the portal vein, was associated with hepatic insulin resistance because the liver is directly exposed to FFAs and cytokines released from the fatty tissue [27–29]. Moreover, the secretion of interleukin (IL)-6 from adipose tissue causes hepatic insulin resistance [30]. The plasma IL-6 levels were 50% higher in the portal vein than in the systemic circulation, and portal vein IL-6 levels were associated with the systemic CRP levels [31]. Indeed, the results from this study showed that the IVAT area index was related to the CRP levels, whereas the RVAT area index was not. Besides, a study demonstrated that hs-CRP levels were associated with visceral fat amount and dysfunction in obese females [32]. On the other hand, adiponectin, leptin, and resistin are associated with insulin resistance [33]. Previous study demonstrated that adiponectin level was inversely related to IVAT, but not associated with SAT, and RVAT, and leptin was associated with all part of adipose tissue [33]. However, there was no association between resistin and adipose tissue distribution [33].

This study has some limitations. First, although a larger number of participants were evaluated than in previous studies that used MRI [11, 12], the relatively small number of the prevalence or incidence of DM limited the number of independent variables that could be included in the multivariate analysis. Therefore, we performed statistical analyses on many models. Second, the study participants were only Japanese men and women, and it is uncertain whether the results can be generalized in other ethnicities. Besides, abdominal CT scanning was performed on request of individuals. This may have introduced an inherent bias in our study design. Finally, CT is a reliable method to evaluate the IVAT or RVAT area [9], but the automated analysis of the IVAT or RVAT area is difficult; therefore, these two parameters were evaluated semi-automatically. Previous studies that used CT, similarly as in this study, indicated that the IVAT or RVAT areas had a high correlation between the first and second manual measurements [9].

In conclusion, the IVAT, but not the RVAT, as evaluated by CT, was associated with the prevalence or incidence of DM. Indeed, IVAT and RVAT might have distinct functions in terms of the prevalence or incidence of DM. Evaluating IVAT and RVAT may enable early detection of prediabetes in obese patients. Further research is required to provide evidence of the effectiveness and feasibility of evaluating IVAT and RVAT.

## Data Availability

Data from this study can be acquired from the corresponding author upon reasonable request.

## Abbreviations

VAT: Visceral adipose tissue
IVAT: Intraperitoneal VAT
RVAT: Retroperitoneal VAT
SAT: Subcutaneous adipose tissue
DSAT: Deep SAT
SSAT: Superficial SAT
CI: Confidence interval
DM: Diabetes mellitus
CT: Computed tomography
CRP: C-reactive protein
FPG: Fasting plasma glucose
LDL-C: Low-density lipoprotein cholesterol
HDL-C: High-density lipoprotein cholesterol
HU: Hounsfield unit
OR: Odds ratio
HR: Hazards ratio
ROC: Receiver operator characteristic
AUC: Area under the ROC curves
FFA: Free fatty acid
